# Calretinin-positive L5a pyramidal neurons in the development of the paralemniscal pathway in the barrel cortex

**DOI:** 10.1186/s13041-014-0084-8

**Published:** 2014-11-18

**Authors:** Junhua Liu, Bin Liu, XiaoYun Zhang, Baocong Yu, Wuqiang Guan, Kun Wang, Yang Yang, Yifan Gong, Xiaojing Wu, Yuchio Yanagawa, Shengxi Wu, Chunjie Zhao

**Affiliations:** Key Laboratory of Developmental Genes and Human Diseases, MOE, Department of Anatomy and Neuroscience, Medical School, Southeast University, Nanjing, 210009 PR China; Center of Depression, Beijing Institute for Brain Disorders, Beijing, 100069 PR China; Institute of Neurobiology, Institutes of Brain Science and State Key Laboratory of Medical Neurobiology, Fudan University, Shanghai, 200032 PR China; Department of Genetic and Behavioral Neuroscience, Gunma University Graduate School of Medicine, 3-39-22 Showa-machi, Maebashi, 371-8511 Japan; Department of Anatomy, Histology and Embryology, K.K. Leung Brain Research Centre, School of Basic Medicine, Fourth Military Medical University, Xi’an, 710032 PR China

**Keywords:** Calretinin, L5a pyramidal neuron, Paralemniscal pathway, Posterior medial nucleus, Barrel cortex

## Abstract

**Background:**

The rodent barrel cortex has been established as an ideal model for studying the development and plasticity of a neuronal circuit. The barrel cortex consists of barrel and septa columns, which receive various input signals through distinct pathways. The lemniscal pathway transmits whisker-specific signals to homologous barrel columns, and the paralemniscal pathway transmits multi-whisker signals to both barrel and septa columns. The integration of information from both lemniscal and paralemniscal pathways in the barrel cortex is critical for precise object recognition. As the main target of the posterior medial nucleus (POm) in the paralemniscal pathway, layer 5a (L5a) pyramidal neurons are involved in both barrel and septa circuits and are considered an important site of information integration. However, information on L5a neurons is very limited. This study aims to explore the cellular features of L5a neurons and to provide a morphological basis for studying their roles in the development of the paralemniscal pathway and in information integration.

**Results:**

1. We found that the calcium-binding protein calretinin (CR) is dynamically expressed in L5a excitatory pyramidal neurons of the barrel cortex, and L5a neurons form a unique serrated pattern similar to the distributions of their presynaptic POm axon terminals.

2. Infraorbital nerve transection disrupts this unique alignment, indicating that it is input dependent.

3. The formation of the L5a neuronal alignment develops synchronously with barrels, which suggests that the lemniscal and paralemniscal pathways may interact with each other to regulate pattern formation and refinement in the barrel cortex.

4. CR is specifically expressed in the paralemniscal pathway, and CR deletion disrupts the unique L5a neuronal pattern, which indicates that CR may be required for the development of the paralemniscal pathway.

**Conclusions:**

Our results demonstrate that L5a neurons form a unique, input-dependent serrated alignment during the development of cortical barrels and that CR may play an important role in the development of the paralemniscal pathway. Our data provide a morphological basis for studying the role of L5a pyramidal neurons in information integration within the lemniscal and paralemniscal pathways.

## Background

In rodents, the primary somatosensory cortex (barrel cortex) integrates into the barrel- and septum-related columnar circuits and receives distinct sensory inputs via the lemniscal and the paralemniscal pathways from the whiskers [1,2]. Via a relay of the principal sensory trigeminal nucleus (Pr5) and the contralateral ventroposteromedial nucleus (VPM), the lemniscal pathway transmits whisker-specific information exclusively to the barrel circuit and forms an input-dependent barrel pattern from the periphery to the center [3]. Likewise, through the spinal trigeminal nucleus (Sp5i) and the contralateral posterior medial nucleus (POm), the paralemniscal pathway transmits information from multiple whiskers into both barrel and septa circuits [1,2,4,5]. The main targets of POm projections are L5a neurons, although the axons also terminate at layer 4 (L4) septa and in L1. It is known that presynaptic POm axon terminals form a distinct pattern in which more axons project to the areas of L5a under septa than to the neighboring areas under barrels, and these terminals display regularly spaced triangular structures in L5A septa columns [1,2,6,7]. However, whether L5a neurons are organized into unique patterns in the paralemniscal pathway, similar to those of L4 neurons in the lemniscal pathway, has not been clearly demonstrated.

In the barrel cortex, L5a is involved in both barrel and septa circuits and is considered to be an important site of information integration for the lemniscal and paralemniscal pathways [4,8]. Electrophysiological studies show that L5a pyramidal neurons are exclusively activated by input from the POm rather than the VPM [4] and can form various types of intracortical synaptic connections with layer 2/3 (L2/3) neurons, L4 spiny satellite cells, and local L5a pyramidal neurons [9-11]. These studies have all indicated that L5a pyramidal neurons play key roles in barrel and septa circuits [4,12]. However, to date, L5a pyramidal neurons are mainly distinguished by their approximate positions or by neuronal tracing, and the morphology of L5a pyramidal neurons remains poorly understood, which has prevented further clarification of the roles of L5a pyramidal neurons in the integration of information.

In this study, we found that the calcium binding protein calretinin (CR) is dynamically expressed in L5a pyramidal neurons of the barrel cortex. L5a pyramidal neurons form a unique, predominantly serrated pattern that is quite similar to the presynaptic projection pattern formed the POm in L5a. This unique alignment is dependent on sensory input, and its organization is synchronous with barrel formation during brain development. Furthermore, deletion of CR disrupts this alignment. In addition to L5a pyramidal neurons, CR is also expressed in the POm and Sp5i of the paralemniscal pathway during development, which indicates that CR may be involved in the development of the paralemniscal pathway. Our results provide an important morphological basis for studying the functions of L5a pyramidal neurons during paralemniscal pathway development and information integration.

## Results

### CR is dynamically expressed in L5a pyramidal neurons of the barrel cortex

**Figure 1:**
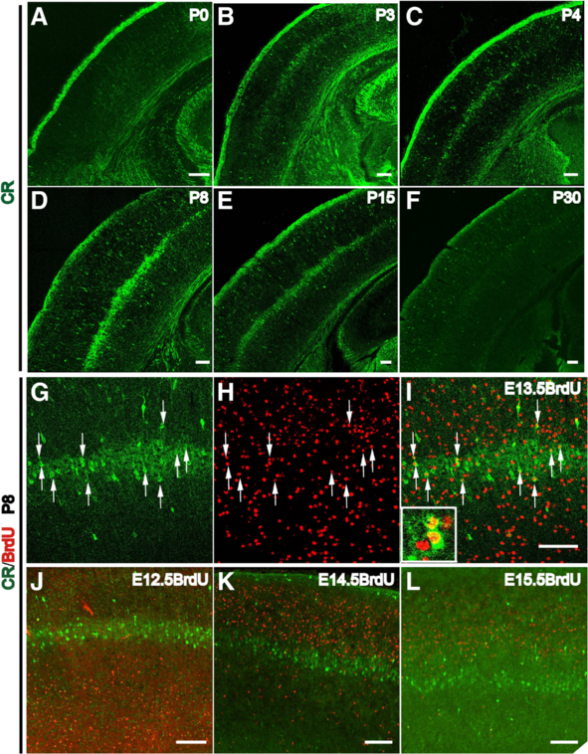
**CR is dynamically expressed in layer 5 of the barrel cortex during the first postnatal weeks.** At birth, no CR staining can be observed in the deeper layer of the cortex **(A)**. Beginning at P3, CR is detected in the deeper layer of the barrel cortex **(B)**. This CR expression gradually increases and forms a clear row-like zone at P4 **(C)**. The expression level of CR peaks at P8 **(D)**. The intensity of the row-like zone of CR staining decreases at P15 **(E)**. By P30, the CR-positive row-like zone can no longer be detected in the deep layer of the cortex, and the only signal comes from CR-positive interneurons dispersed throughout the cortex **(F)**. Birth-dating analysis indicates that abundant CR-positive neurons at P8 are found to be positive for BrdU administered at E13.5 (**G**, **H**, **I**, arrows). Few BrdU- and CR-positive neurons were observed when BrdU was injected at E12.5 **(J)**, E14.5 **(K)** and E15.5 **(L)**, which corresponds to the time points at which L6, L4 and L2/3 neurons, respectively, are born. Scale bar: 100 μm.

CR, a calcium-binding protein, is reported to be an important modulator of neuronal excitability [13], with involvement in synaptogenesis, axonal elongation and dendritic remodeling [13,14]. Here, we detected the postnatal expression and possible role of CR in the mouse somatosensory cortex. At birth, CR was scarce in the somatosensory cortex (Figure 1A). At P3, a row-like band of CR expression could be detected in the deep layer of the barrel cortex (Figure 1B), and the expression level of CR gradually increased until it peaked at P8 (Figure 1D), as previously reported [14]. Scattered CR-positive neurons throughout the cortex were also observed (Figure 1A-F), and these neurons are likely interneurons according to previous reports [15,16]. We further found that neurons that strongly expressed CR in the row-like zone within the deeper layer of the barrel cortex are horizontally aligned in a serrated pattern (Figure 1C-D). After P8, the expression level of CR then gradually decreased and barely detectable at P30, except in scattered interneurons (Figure 1E-F). This dynamic expression of CR in the deeper layer of the barrel cortex is consistent with the time window during which cortical barrels develop, which suggests a possible role for CR in barrel cortex development. To identify the features and functions of the CR-positive cells, we first performed a BrdU birth-dating analysis. BrdU was injected at E12.5, E13.5, E14.5, E15.5, respectively, corresponding to the time points when layer 6 (L6), L5 and upper layer neurons are born. The brains were then harvested at P8, when the expression of CR is strongest in the deeper layer in the barrel cortex and almost all of the projection neurons had migrated to their final destinations in the cortical plate. Double immunostaining for CR and BrdU revealed that approximately 43.40 ± 2.70% of neurons with strong CR expression in the row-like zone were also positive for BrdU administered at E13.5, which is the time point when the majority of L5 neurons are generated (Figure 1G,H,I, arrow and insert). But few of the strongly CR-positive neurons were positive for BrdU labeling applied at other time points (Figure 1J,K,L), suggesting that CR-positive neurons are mainly born at E13.5.

**Figure 2:**
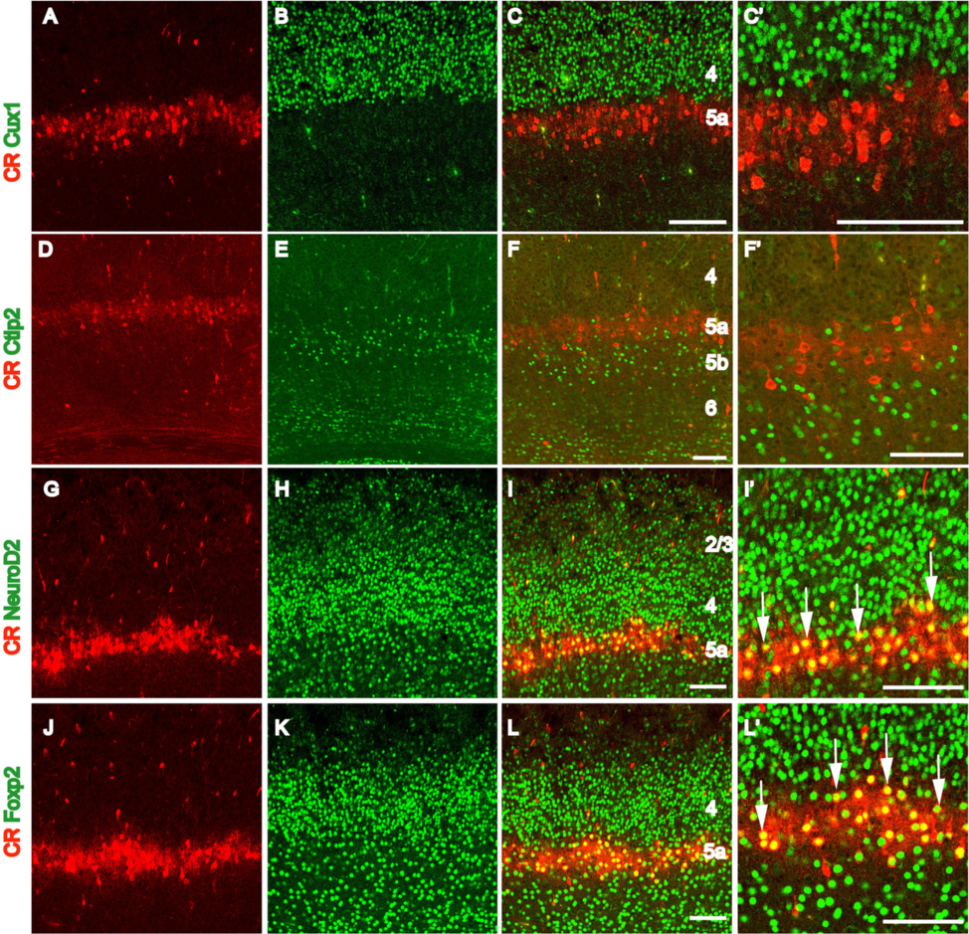
**CR-positive neurons are L5a excitatory pyramidal neurons.** Cux1 is specifically expressed in the upper layer (L2/3 and L4) excitatory neurons **(B)**. CR-positive neurons are located just below the Cux1-positive neurons **(A, C, C’)**, and no CR- and Cux1-positive neurons are observed **(C, C’)**. Ctip2 is strongly expressed in L5b excitatory neurons **(D)**. CR-positive neurons are located above the Ctip2-positive L5b neurons **(E, F, F’)**, and no CR- and Ctip2-positive neurons are observed **(F, F’)**. The CR-positive neurons are located between the Cux1-positive L2-4 neurons and the Ctip2-positive L5b neurons within the region corresponding to L5a **(C’, F’)**. Most of the CR-positive neurons are NeuroD2- and Foxp2-positive **(G-** **L’**, arrows). Scale bar: 100 μm.

In the barrel cortex, L5 consists of two populations of L5a and L5b neurons that receive paralemniscal and lemniscal projections, respectively. We further determined the exact localization of CR-positive neurons within L5 at P8. When slices were co-labeled with Cux1, an upper layer (L2/3 and L4) excitatory neuronal marker, and Ctip2, a marker for L5b excitatory neurons [17,18], CR-positive neurons were found between the layers of the Cux1-positive and L5b Ctip2-positive neurons without co-localization with either marker (Figure 2A-F’). Meanwhile, two transcription factors that are widely expressed in cortical excitatory neurons, neurogenic differentiation 2 (NeuroD2) [19] and forkhead box proteins 2 (Foxp2) [20], were found to be expressed in most of the CR-positive neurons (Figure 2G-L’). Statistical analysis revealed that in the row-like zone, 96.22 ± 2.94% of CR-positive neurons were NeuroD2-positive and 97.04 ± 2.55% were Foxp2-positve. Taken together, our data indicate that CR-positive neurons are L5a excitatory pyramidal neurons, the main target of POm projections [4].

**Figure 3:**
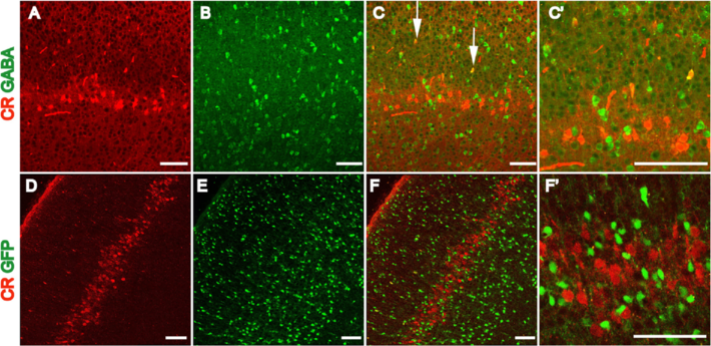
**CR-positive neurons in L5a are not GABAergic interneurons.** Numerous GABA-positive interneurons are detected in the barrel cortex at P8 **(B, C)**, but none of these neurons are positive for CR in L5a **(A-C’)**. CR- and GABA-positive interneurons can be observed in other areas of the cortex (**C**, arrows). In the GAD67-GFP transgenic cortex, abundant GFP-positive interneurons are detected, but none of these neurons are positive for CR in L5a **(E, F, F’)**. Scale bar: 100 μm.

To exclude the possibility that CR-positive neurons in L5a are interneurons, double immunostaining for anti-CR and anti-gamma-aminobutyric acid (anti-GABA) was performed. Within the row-like zone, no CR-positive neurons were found to also be GABA-positive, although CR- and GABA-positive interneurons were found scattered throughout the cortex (Figure 3C,C’). This result was also confirmed by co-labeling CR with anti-GFP using the GAD67-GFP knock-in mouse line in which GFP is expressed in almost all GABAergic interneurons under the control of the endogenous GAD67 promoter [21]. Abundant GFP-positive interneurons were detected throughout the cortex, but few GFP-positive interneurons within L5a were found to also be CR-positive (Figure 3F,F’). These data all indicate that CR-positive neurons within L5a are excitatory pyramidal neurons and not GABAergic interneurons.

### L5a pyramidal neurons form a unique serrated pattern that requires sensory input during early postnatal days for maintenance

**Figure 4:**
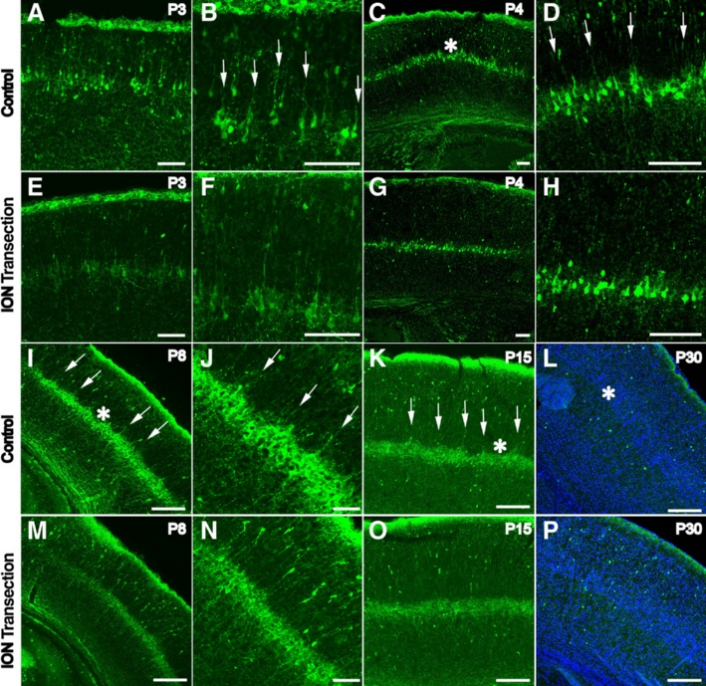
**Maintaining the distinct serrated alignment of CR-positive L5a neurons requires sensory input.** CR-positive neurons in L5a send long processes into L4 at P3 (**A**, **B**, arrows). Until P4, the CR-positive L5a neurons display the distinct serrated aligning pattern, and their processes form septa-like structures **(C, D)**. At P8, the septa-like structures become more distinct **(I, J)**. The expression pattern of CR is maintained until P15 **(K)**. At P30, the CR expression in L5a disappears, and the barrels in L4 become more distinct. After ION transection at P2, the expression level of CR rapidly declines at P3 **(E, F)**. From P4 to P15, the processes of the CR-positive neurons become shorter; the distinct serrated aligning pattern is disrupted; and no septa-like structures of the CR-positive processes are observed **(G, H, M, N, and O)**. By P30, the expression of CR in L5a disappears, and only scattered CR-positive neurons are observed throughout the cortex **(P)**. The asterisks in **C**, **I** and **L** denote barrels and prospective barrels. The arrows in **B**, **D**, **J**, and **K** denote processes that extend to L4 to form septa-like structures. Scale bar: 100 μm.

In the barrel cortex, L5a pyramidal neurons specifically receive inputs from the POm [1,2,4,7], whose axon terminals display a row-like pattern with regularly spaced triangular structures in L5a. It has been reported that the POm projects more axons to L5a neurons vertically aligned under septa than to the adjacent neurons below the barrels [1,2,7]. This distinct projection pattern of the POm afferents may recruit their postsynaptic partner L5a pyramidal neurons to form a projection-related distribution pattern. To test this hypothesis, a detailed analysis of the distribution of CR-positive L5a neurons was performed by immunostaining at various time points during development of the barrel cortex. At P3, the radial processes of L5a neurons started to extend into the overlying L4 (Figure 4A,B). At P4, CR was observed to be expressed in more L5a neurons and formed a clear row-like structure (Figure 4C). Furthermore, we found that the CR-positive L5a neurons displayed a non-homogeneous distribution with some cells aggregated into serrated structures, and the pattern was quite similar to that of their presynaptic POm projections (Figure 4C,D). Regularly spaced CR-positive processes were observed to extend from the serrated structures into L4 (Figure 4D). The expression of CR peaked at P8 (Figure 4I), and the serrated distribution pattern became more defined, with the neuropil spanning all of L4 and forming a prominent septa-like pattern (Figure 4J). The septa-like pattern could be observed until P15, but the expression level of CR in L5a decreased from P8 on (Figure 4K). The structures between the septa-like CR-positive processes might represent prospective barrels (Figure 4I-K). By P30, the CR-positive row-like structure with septa-like processes had almost disappeared, and the barrel structures became more apparent (Figure 4L). These observations suggested that L5a neurons could be recruited by inputs from the POm to form serrated structures with their processes extending to L4 as septa-like structures that closely resembled the projection pattern of the POm into L5a.

It has been reported that the first postnatal week is critical for experience-dependent pattern formation in the barrel cortex. The afferent signal is the principal element in experience-dependent neuronal patterning [3,22,23]. It is possible that the distribution pattern of CR-positive L5a neurons may also be input-dependent. To further detect whether the distribution pattern of CR in L5a depends on sensory input, an early transection of the infraorbital nerve (ION) experiment was carried out. ION transection at P2 led to markedly reduced expression of CR in the neuropil and cell bodies of the L5a neurons by P3 (Figure 4E,F). By P4, the CR-positive processes that extend to L4 had become shorter, and the septa-like pattern disappeared (Figure 4G,H). At P8 and P15, few septa-like CR-positive processes were observed to extend to L4. More interestingly, the serrated distribution pattern of CR-positive L5a neurons also disappeared, and only the uniform row-like CR-positive zone remained (Figure 4M,N,O). By P30, expression of CR in L5a could no longer be detected, similar to observations of the control. Taken together, our data indicate that the unique serrated alignment of CR-positive L5a pyramidal neurons is related to the paralemniscal pathway and depends on peripheral sensory input.

### The alignment of L5a neurons develops synchronously with formation of the cortical barrels

Timing of the formation of the distinct serrated alignment of L5a neurons corresponds to the time window of cortical barrel formation and is input dependent, suggesting that the lemniscal and paralemniscal pathways might develop synchronously. We next compared the temporospatial development of the neuronal pattern of L5a with that of the barrels in L4 within the barrel cortex.

**Figure 5:**
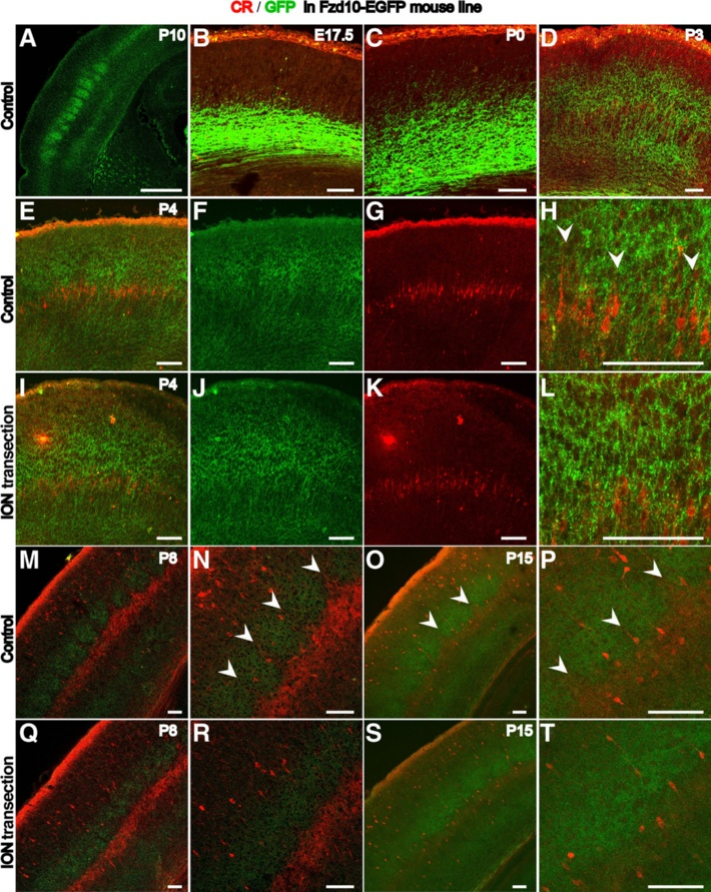
**The alignment of L5a neurons develops synchronously with the formation of cortical barrels.** Specifically labeled axons of the VPM in the Fzd10-EGFP transgenic line **(A)**. At E17.5, GFP-positive TCAs from the VPM extend into the deep cortical layer **(B)** and reach the superficial layer of the barrel cortex at P0 **(C)**. No expression of CR is detected in the barrel cortex except in the Cajal–Retzius cells under the pia. At P3, the GFP-positive TCAs segregate into two strong GFP-positive bands in layer 4 and layer 6, and CR-positive neurons emerge in L5a **(D)**. At P4, the GFP-positive TCAs of the VPM begin to segregate into clusters but do not completely separate from each other **(E, F)**. The CR-positive processes extend into L4 and show a septa-like pattern (**G**, **H** arrowheads). Until P8, the GFP-positive clusters in L4 and the CR-positive L5a neurons display a complementary distribution pattern **(M, N)**. Between L4 and L5a, the CR-positive L5a neurons display a distinct serrated alignment, and their processes extend vertically between the GFP-positive clusters (**N**, asterisk). At P15, the complementary pattern of the TCAs from the VPM and the CR-positive L5a neurons remain evident, but the expression level of CR is decreased (**O**, **P**, arrowhead). After ION transection at P2, no septa-like pattern can be observed at P4 **(K, L)**, and the GFP-positive TCAs are not segregated into clusters **(I, J)**. Only uniform GFP-positive and CR-positive bands remain in L4 and L5a, respectively, and no barrel-like or septa-like pattern can be observed at P8 **(Q, R)** and P15 **(S, T)**. Scale bar in (A): 500 μm, Scale bar in other images: 100 μm.

Previously, we reported a transgenic mouse line Fzd10-TauLacZ in which the thalamocortical afferents (TCAs) from the VPM are specifically labeled [24]. We used the same Fzd10 promoter to generate an EGFP reporter mouse line (Figure 5A). Using this mouse line, we found that the GFP-positive TCAs had already reached the deep layer in the barrel cortex at E17.5 (Figure 5B) and had extended their arbors into L4 by P0 (Figure 5C). However, at P0, no CR expression was detected in L5a (Figure 5C). By P3 and P4, the GFP-positive TCA terminals had invaded L4 (Figure 5D,E,F) and segregated into clusters, but the clusters were not completely separated from each other (Figure 5E,F). Meanwhile, CR expression was observed in L5a, and some of the CR-positive neuropil had begun to extend into the overlying L4 (Figure 5D,G,H). At P8, the GFP-positive TCA terminals in L4 were completely segregated into distinct clusters to form barrels (Figure 5M,N). At the boundary of L4 and L5, the CR-positive L5a pyramidal neurons intruded into regions between the TCA terminal clusters and formed regularly spaced triangular alignment structures from which the CR-positive neuropil extended into L4 to form a septa-like pattern (Figure 5M,N). The alignment of the CR-positive L5a neurons complemented this pattern of GFP-positive TCA terminals. From P8 to P15, when the barrels were nearly mature, the GFP-positive TCA clusters became denser, and the expression intensity of CR decreased (Figure 5O,P). We then performed ION lesions at P2 and found that the unique alignment of CR-positive neurons was disrupted and that the CR-positive neuropil became shorter. Although the GFP-positive TCAs were able to reach L4, no barrels formed. The serrated pattern of the L5a neurons disappeared; instead, a uniform CR-positive band was detected, and no septa-like pattern was observed (Figure 5K-L, Q-T). These results suggest that segregation of the VPM projections and formation of the CR-positive L5a pattern may occur in parallel during development of the cortical barrels.

### The serrated alignment of L5a neurons is disrupted in CR mutants

**Figure 6:**
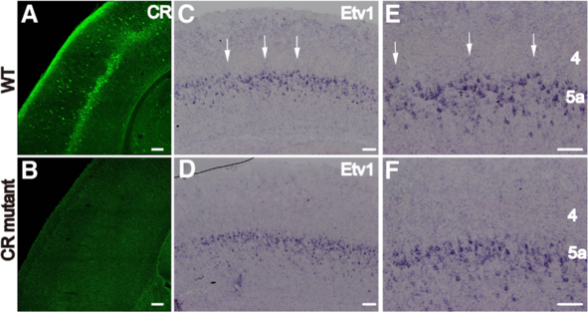
**The distinct serrated alignment of L5a pyramidal neurons is disrupted in the CR KO cortex.** CR is effectively deleted in the CR mutant cortex compared to that of the WT at P8 **(A, B)**. In situ hybridization for the transcription factor Etv1, which is mainly expressed in the L5a neurons, reveals a prominent serrated alignment with regularly spaced triangular structures protruding towards the overlaying layer in WT animals (**C**, **E**, arrows) that is disrupted in the mutants **(D, F)**. Scale bar: 100 μm.

The distinct expression pattern of CR in L5a neurons raised the possibility that CR may be important for development of the paralemniscal pathway. As a member of the calmodulin superfamily, CR plays an important role in the precise regulation of intracellular calcium signals and neuronal excitability [13,25-27]. Therefore, we next examined the alignment of L5a neurons in CR knockouts [28]. In situ hybridization was performed to detect the distribution of the ets variant 1 (Etv1) gene, which is a transcription factor reported to be specifically expressed in L5a neurons [29,30]. At P8 in controls, Etv1-positive L5a neurons displayed a distinctive serrated pattern with regularly spaced triangular structures protruding towards the overlaying layer that corresponded to the pattern of CR (Figure 6C and E, arrows). However, in CR mutants, the serrated pattern was disrupted with no triangular structures and a uniform distribution of Etv1-positive L5a neurons (Figure 6D-F), indicating that CR is important for the formation of the distinct serrated arrangement of L5a pyramidal neurons.

### CR is also specifically expressed in the relay nucleus of the paralemniscal pathway

**Figure 7:**
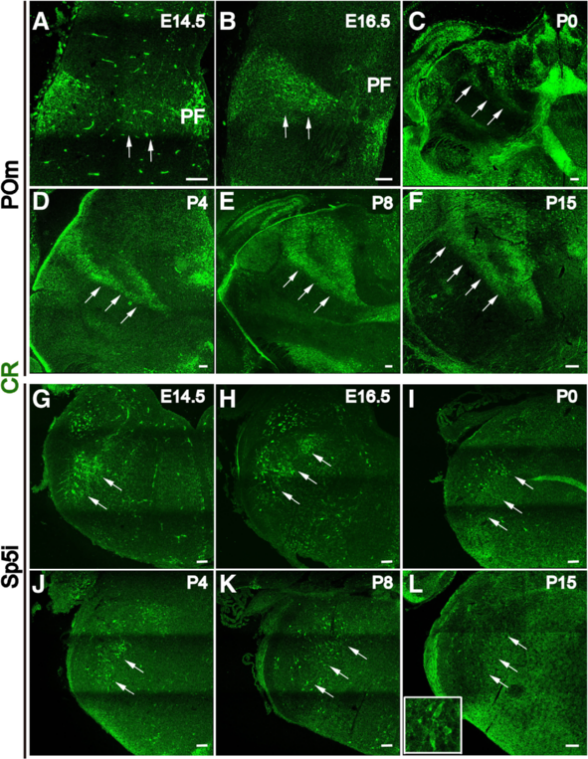
**CR is strongly expressed in the developing paralemniscal pathway.** At E14.5, weak CR can be observed in the POm, lateral to the parafascicular thalamic nucleus (**A**, arrows). At E16.5, the expression of CR in the POm becomes stronger (**B**, arrows). After birth, CR is continuously expressed in the POm at least through P15 (**C-F**, arrows). Strong expression of CR is also observed in Sp5i, which is the relay nucleus of the paralemniscal pathway, from E14.5 to P15 (**G-L**, arrows). Scale bar: 100 μm.

**Figure 8:**
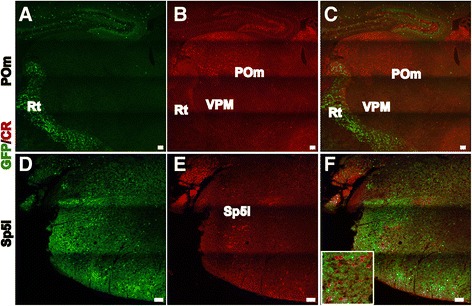
**CR-positive neurons in the Sp5i and POm are not GABAergic interneurons.** Double immunolabeling of anti-CR and GFP in GAD67-GFP mice at P8 indicates that GFP-positive neurons are located in the reticular thalamic nucleus (Rt) and are not co-labeled with CR **(A)**. CR expression is specifically detected in the POm in the dorsal thalamus **(B, C)**. In the medulla oblongata, CR-positive neurons and GFP-positive neurons can be detected in the Sp5i, but CR and GFP are not co-labeled **(D-F)**. The insert represents an amplification of CR-positive neurons in the Sp5i. Scale bar: 100 μm.

The paralemniscal pathway originates from large neurons in the interpolar subnucleus of the spinal trigeminal nucleus (Sp5i), which project to the POm in the thalamus [31], and these POm afferents target L1, L5a and the septum-related L4 neurons in the S1 cortex. We found that, in addition to being expressed in L5a pyramidal neurons, CR was also dynamically expressed in the POm and Sp5i, which are the relay nuclei of the paralemniscal pathway. At E14.5, the expression of CR could be observed in the POm, which is located lateral to the parafascicular thalamic nucleus (PF) (Figure 7A) and the Sp5i (Figure 7G). This expression became stronger at E16.5 (Figure 7B,H) and continued to persist (Figure 7C-F,I-L). To determine whether CR-positive neurons are excitatory neurons or interneurons, double labeling of anti-CR and GFP was performed in the GAD67-GFP knock-in mouse line at P8, when the pattern of the paralemniscal pathway had formed in L5a. No co-localization was detected in the POm or Sp5i (Figure 8A-F, insert), which indicates that these CR-positive neurons are excitatory projection neurons. The expression of CR in the developing paralemniscal pathway suggests that CR may be involved in development and maturation of the paralemniscal pathway as a modulator of neuronal excitability and of calcium signaling pathways.

## Discussion

In rodents, the barrel cortex is a unique columnar neuronal circuit with barrel and septa columns (circuit) that receive distinct sensory information through the lemniscal and the paralemniscal pathways from the VPM and POm, respectively [1,2,5]. Information from both circuits must be integrated in the barrel cortex to mediate precise object recognition and related behavioral tasks [1]. It has been reported that barrel dysfunction is associated with Fragile X mental retardation syndrome [32] and Huntington’s disease [33]. Therefore, it is crucial to understand the mechanisms underlying the development of lemniscal and paralemniscal pathways, as well as the integration of information in the whisker-barrel cortex.

In the barrel cortex, L5a pyramidal neurons are of particular interest not only because they receive subcortical inputs exclusively from the paralemniscal pathway but also because they are involved in both the barrel and septa circuits [4,9,11,12]. L5a pyramidal neurons are considered to be an important integration site of the lemniscal and paralemniscal pathways and the key output layer of the barrel cortex [5,10,12,34]. However, information about L5a pyramidal neurons has remained limited.

### The unique alignment of L5a pyramidal neurons may form part of the morphological basis for information integration in the barrel cortex

It is well known that the formation of peripheral-input-related neuronal patterning in the barrel cortex depends on thalamocortical afferents. Previous studies have demonstrated that the neuronal pattern in the lemniscal pathway in L4 is organized only after segregation of the presynaptic afferents [3,22]. However, it remains unclear whether L5a neurons in the paralemniscal pathway integrate into an input-dependent distribution pattern during development that resembles the pattern of L4 neurons. Using retrograde neuronal tracing, POm axons have been shown to project mainly to L5a. Projections of the POm to L5a neurons in septa columns are denser than those to their neighbors just below the barrel columns, and POm projections form a row-like pattern with regularly spaced triangular structures, indicating that there may be a postsynaptic paralemniscal input-related neuronal pattern in L5a. In this study, using CR as a specific marker for L5a pyramidal neurons, we determined that L5a neurons are organized in a serrated alignment pattern complementary to the distribution pattern of the POm projection terminals. Furthermore, the disruption of this unique alignment after ION transection suggests that it is input dependent. Taken together, in the barrel cortex, there is an input-dependent neuronal pattern in L5a neurons of the paralemniscal pathway comparable to that formed by L4 neurons in the lemniscal pathway.

Although L5a pyramidal neurons are the main targets of the paralemniscal pathway, they also receive indirect lemniscal pathway input through monosynaptic connections between L4 barrel neurons and L5a neurons [9], as well as intracolumnar input from L2/3 and L5b in barrel columns [8,35]. L5a neurons also send weak outputs to L2/3 neurons in the barrel columns [4,9,10,35,36]. In addition, L5a pyramidal neurons form local connections [11], sending outputs to intracolumn L5b cells [37], intercortical motor and secondary somatosensory cortices [5,10,34], and the basal ganglia [38,39]. Electrophysiological studies have unanimously indicated the importance of L5a pyramidal neurons as the integration site of the lemniscal and paralemniscal pathways. Feldmeyer, Roth and colleagues have reported synaptic connections between L4 spiny stellate cells and L5a pyramidal cells, and postsynaptic L5a somata are mostly located under the barrel wall and septa, suggesting the existence of a short circuit between the lemniscal and paralemniscal pathways [9]. They also found local connections in paired L5a pyramidal neurons, mainly along the lateral border of barrel columns, and these connections appear to form vertical clusters. It is plausible that the vertical clusters may correspond to the extrusions within the serrated pattern of L5a pyramidal neurons observed in this study. Our observations have provided morphological support for connections between the barrel and septa columns. Meanwhile, more attention should be paid to the L5a neuronal intrusions into the lower part of L4 for further studies of the paralemniscal-related neuronal circuit and the integration of information in the barrel cortex.

### The long processes of CR-positive L5a neurons may contribute to the composition of septa

In this study, we have shown that development of the L5a neuronal pattern parallels the segregation of VPM axons during the time window of barrel formation, indicating that the lemniscal and paralemniscal pathways may interact to regulate pattern formation and refinement of the barrel cortex.

In the somatosensory cortex, L4 is organized into cell-dense barrels and cell-sparse septa [40], which receive thalamocortical input from the VPM and POm, respectively [1,2]. However, because septa are small and contain few cells, their properties remain largely unknown. In this study, from intrusions of L5a neuron alignment, we directly observed that the CR-positive neuropil extends into L4 and forms a septa-like pattern, which suggests that the dendrites of L5a pyramidal neurons may be components of the septa. Further studies are needed to understand the connections of septa with L5a pyramidal neurons and to help elucidate the function of septa and the septal circuit.

### CR may be involved in development of the paralemniscal pathway and formation of the L5a neuronal alignment

The dynamic expression of CR in L5a neurons while cortical barrels are developing and the disruption of the distinct alignment of L5a pyramidal neurons in CR-null mutants indicate that CR, a critical element of calcium signaling, is involved in the development and maintenance of input-dependent patterning in L5a pyramidal neurons. Furthermore, the specific expression of CR in the paralemniscal pathway indicates that CR may also be required during development of the paralemniscal pathway and may therefore be important for its function. In this study, we were unable to evaluate the electrophysiological characteristics of CR-deficient L5a neurons. Further studies using CR mutants will be useful to elucidate the mechanisms underlying development of the paralemniscal pathway and its interaction with the lemniscal pathway in the barrel cortex.

## Conclusions

In this study, we have shown that, as a main target of the paralemniscal pathway in the barrel cortex, CR-positive L5a pyramidal neurons form the POm projection-related neuronal pattern during early postnatal development, synchronously with lemniscal-related barrel formation. The expression of CR in the paralemniscal pathway and the changed patterning of the L5a neurons in the CR mutant indicated that CR may be important for development of the paralemniscal pathway and the barrel cortex. Our data provide an important morphological basis for studying the functions of L5a pyramidal neurons and development of the paralemniscal pathway in the barrel cortex.

## Methods

### Animals

The generation of GAD67-GFP knock-in mice has been previously described [21]. The Calretinin knockout line [28] was purchased from Jackson Laboratory. The Fzd10-EGFP transgenic line was generated using the Frizzled10 promoter to drive the reporter gene as previously reported [24]. Both male and female animals were used in this study. The day the vaginal plug was detected was designated as embryonic day 0.5 (E0.5). The day of birth was designated as postnatal day 0 (P0). Each group subjected to specific examination included at least 3 mice. All of the animals were bred in the animal facility at Southeast University. All of the experiments were performed according to the guidelines approved by Southeast University.

### ION transection

The pups were anesthetized by hypothermia at P2. Under a stereoscopic microscope, the mystacial whiskers on the left side were plucked before the operation. A small incision was made just behind the left whisker pad. The infraorbital nerve was exposed and transected with ophthalmic forceps. After hemostasis and saturation, the pups were put on a heating blanket to recover and returned to their home cages. The pups were then harvested at P3, P4, P8, P15 and P30, respectively. Each time point contained at least 5 pairs of animals.

### Tissue preparation

Mice up to P4 were anesthetized by hypothermia. Mice at P8, P15, and P30 were anesthetized with sodium pentobarbital (50 mg/kg body weight) via intraperitoneal injection. Each anesthetized mouse was perfused transcardially with 0.1 M phosphate-buffered saline (PBS, pH 7.4) followed by 4% paraformaldehyde (PFA) in 0.1 M PBS under the stereoscopic microscope. The brains were removed, post-fixed for 8–10 hours in 4% PFA and protected with 30% sucrose in 0.1 M PBS overnight at 4°C. The brains were sectioned in the coronal plane using a freezing microtome (Leica). The thicknesses of the sections were 16 μm (up to P4) and 25 μm (P8, P15 and P30).

## Immunostaining

Immunostaining was performed according to standard protocols [41]. Briefly, the sections were permeabilized in PBS containing 0.1% Triton X-100 (PBT) for 30 min, blocked with 10% calf serum in PBT for 2 h, incubated at 4°C with primary antibody overnight, then incubated with the secondary antibody for 2 h at room temperature (RT). Slices were then counterstained with the fluorescent nuclear stain DAPI and mounted under coverslips. The sections were washed with 0.1 M PBS between the staining steps.

### BrdU birth-dating

BrdU (Sigma-Aldrich, B5002) was dissolved in physiological saline at a concentration of 10 mg/ml. Pregnant mice were given BrdU (50 mg/kg, intraperitoneal injections) at E12.5, E13.5, E14.5, E15.5, and brains of the offspring were harvested at P8 for birth-dating analysis. CR immunostaining was carried out first, and sections were re-fixed with 4% PFA for 20 min, then treated with 2 N HCl for 15 min at 42°C. After HCl was washed out with PBS, immunostaining for BrdU was applied according to the standard procedure described above. At least three brains were collected from independent litters at each time point.

### Primary and secondary antibodies

The following antibodies were used: rabbit polyclonal anti-CR (Chemicon, AB5054, 1:1000), mouse anti-CR (Chemicon, MAB1568, 1:1000), rat polyclonal anti-BrdU (Abcam, ab6326, 1:1000), rabbit polyclonal anti-Cux1 (Santa Cruz, sc-13024, 1:500), rat polyclonal anti-Ctip2 (Abcam, ab18465, 1:1000), rabbit polyclonal anti-Foxp2 (Abcam, ab16046, 1:1000), rabbit polyclonal anti-NeuroD2 (Abcam, ab31938, 1:100), rabbit polyclonal anti-GABA (Chemicon, AB131, 1:500), chicken anti-GFP (Invitrogen, A10262, 1:500). FITC goat anti-mouse IgG (Jackson ImmunoResearch, 115-095-146, 1:200), Alexa Fluor 555 donkey anti-mouse IgG (Invitrogen, A31570, 1:500), Alexa Fluor 633 goat anti-mouse IgG (Invitrogen, A21050, 1:500), Alexa Fluor 488/633 goat anti-rabbit IgG (Invitrogen, A11008/A21071, 1:500), Alexa Fluor 550 goat anti-rat IgG (Invitrogen, A11006, 1:500), and Alexa Fluor 488 conjugated AffiniPure donkey anti-chicken IgG (Jackson ImmunoResearch, 1:1000). DAPI was purchased from Sigma-Aldrich (D9564).

### Microscopic imaging

Sections were imaged under a confocal microscope (FV1000, Olympus) using the 405, 488, 559, and 633 laser channels. Pictures were captured sequentially for each channel under the 10×, 20× and 40× objectives with excitation lasers and detection spectra calibrated to minimize channel crosstalk. The image format was 1024 × 1024 pixels. Confocal image stacks were analyzed using FV10-ASW 4.1 Viewer.

### In situ hybridization

Brains from P8 mice were perfused with 4% PFA, postfixed in 4% PFA overnight, and cryoprotected in 30% sucrose/DEPC-PBS at 4°C. Coronal sections (25 μm thick) were sliced with a Leica CM 3050S cryostat and stored at −70°C until use. For Etv1 probe preparation, total RNA was extracted from E14.5 brains. Using primers with the sequences GAG TCA CAG GCC AGT AGC ATT and ACA GCA GCG GAA GCA TTA G, we amplified Etv1 fragments that were approximately 421 bp in length (2380–2801), and these fragments were subcloned into pBluescript SK vectors and linearized (details available upon request). In situ hybridization was performed as described previously [41]. Pictures were captured with an OlympusBX61 microscope (Tokyo, Japan). Three brain pairs from three independent litters were examined.

### Cell counting

Coronal sections at three comparative levels from the anterior to posterior in each brain were examined. Pictures were acquired using an Olympus FluoView FV1000 confocal microscopy with the 20× objective lens. Cell counting was performed on sections using image Pro Plus software (Media Cybernetics).

## Abbreviations

ION: Infraorbital nerve
Pr5: Principal sensory trigeminal nucleus
VPM: The contralateral ventroposteromedial nucleus of the thalamus
Sp5i: The interpolar subnucleus of the spinal trigeminal nucleus
POm: The contralateral posterior medial nucleus
L4: Layer 4
P4: Postnatal day 4
E12.5: Embryonic day 12.5
CR: Calretinin
Cux1: Cux family transcription factor 1
Ctip2: Chicken ovalbumin upstream promoter transcription factor-interacting protein 2
NeuroD2: Transcription factor neurogenic differentiation factor 2
Foxp2: Forkhead box protein 2
GABA: Gamma-aminobutyric acid
GAD67: 67-kDa glutamic acid decarboxylase
GFP: Green fluorescent protein
Fzd10: Frizzled10
EGFP: Enhanced green fluorescent protein
TCAs: Thalamocortical afferents
ETV1: Ets variant 1
PF: Parafascicular thalamic nucleus
Rt: Reticular thalamic nucleus
